# Supplementary material for: PIP2 modulates TRPC3 activity via TRP helix and S4-S5 linker
Source: Nat Commun. 2024 Jun 18;15:5220. doi: 10.1038/s41467-024-49396-6 (PMC11189476; doi:10.1038/s41467-024-49396-6)
Supplement: Supplementary file 1 — Supplementary Information [file 41467_2024_49396_MOESM1_ESM.pdf]

Supplementary Information

PIP_2 modulates TRPC3 activity via TRP helix and S4-S5 linker

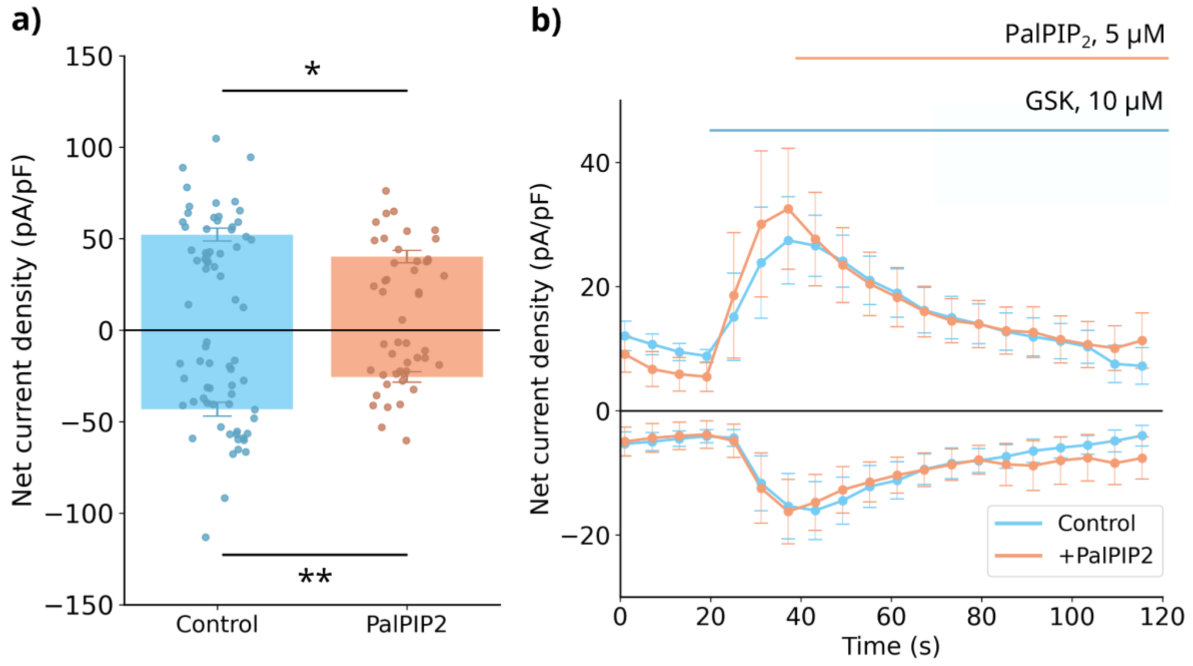

Supplementary Figure 1. Sequestering PIP_2 reduced TRPC3 activity with prolonged, but not with rapid PalPIP2 application. a) Whole-cell net current density recorded from a HEK cell expressing TRPC3 WT, in response to repeated applications of GSK (10 µM) at membrane potentials of −90 mV and +70 mV. Cells were incubated in ECS containing either DMSO (0.01%; Control; blue bar) or PalPIP2 (5 µM in 0.01% DMSO; PalPIP2; orange bar) for 10 min prior to the experiments. Data presented as Mean±SEM (n = 24-36 cells). Statistical significance was assessed using a two-tailed t-test with Bonferroni correction. **P< 0.01, *P< 0.05. b) Time course of current development recorded from a HEK cell expressing TRPC3, in response to repeated applications of GSK (10 µM) at membrane potentials of −90 mV and +70 mV. At the 20-second time point, GSK (10 µM) was applied, and either DMSO (Control; blue line) or PalPIP2 (+PalPIP2; orange line) was subsequently added at the peak of the current. Data presented as Mean±SEM (n = 10-12 cells).

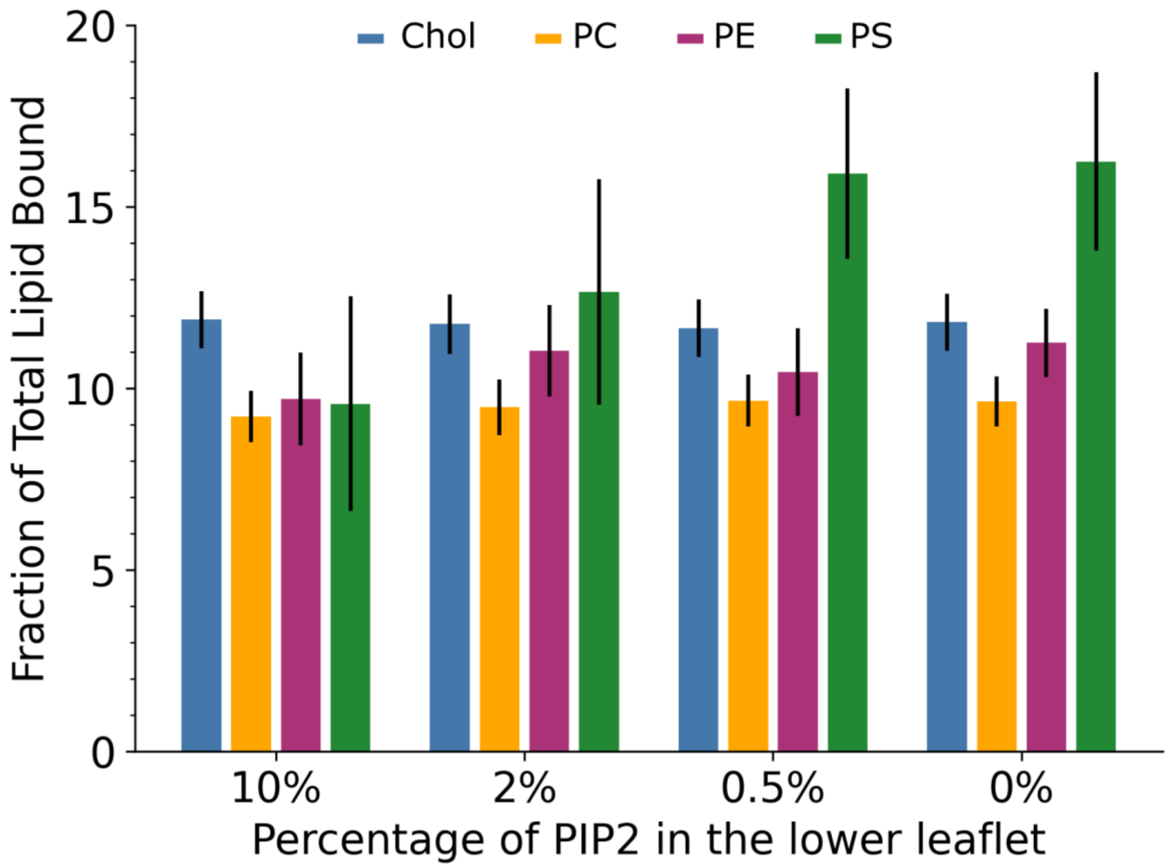

Supplementary Figure 2: Fraction of cholesterol and phospholipids bound to TRPC3. Data is shown for simulations containing 10%, 2%, 0.5% and 0% PIP_2 in the lower leaflet. Fraction bound is calculated as the number of each lipid type bound to TRPC3 divided by the total number of each lipid type present in the simulation. Data is averaged over the equilibrated portion of the simulations (5 to 20 µs). Error bars represent standard deviation. Each set of simulations consists of 5 repeats.

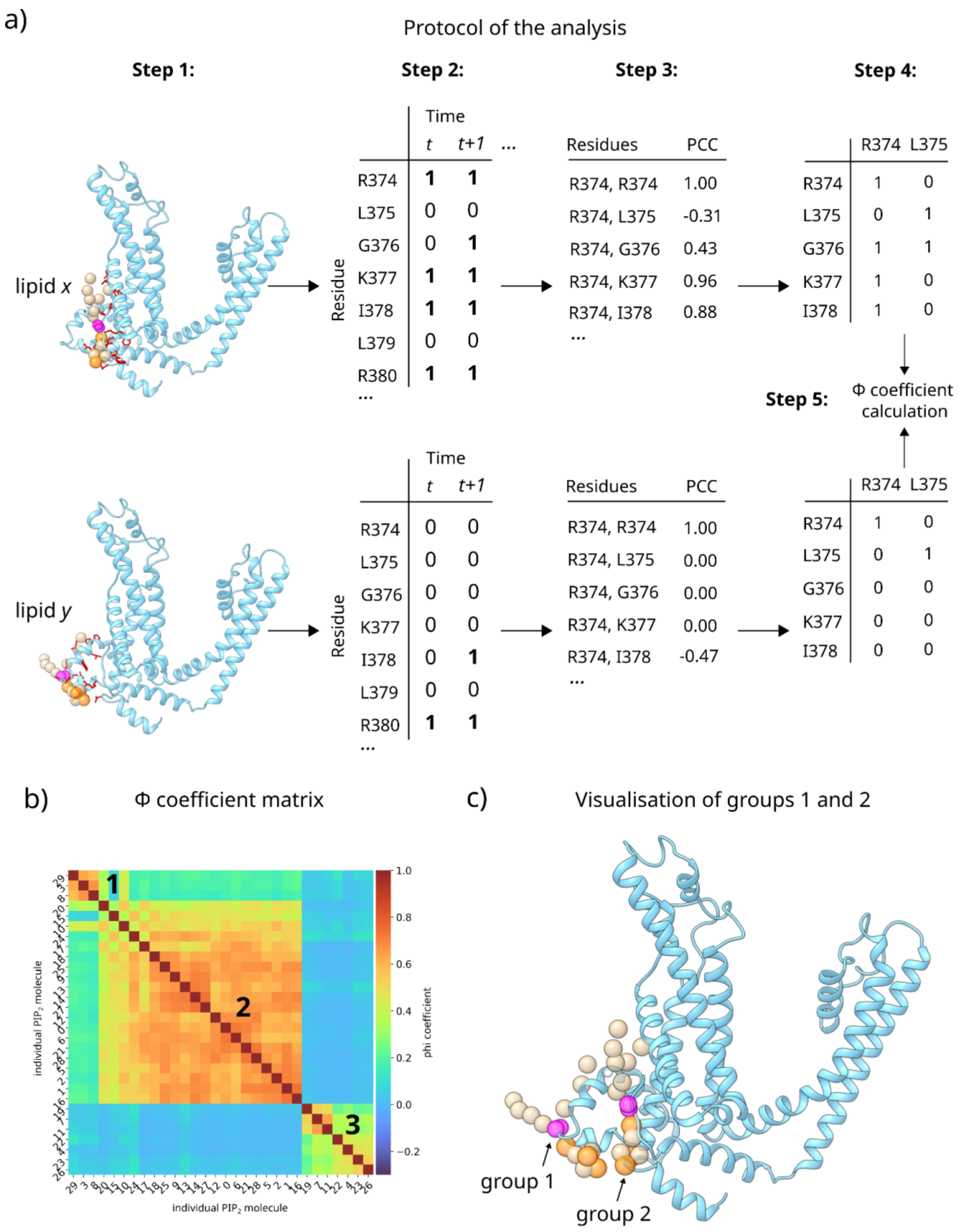

Supplementary Figure 3: Clustering of PIP_2 binding events shows that the majority of PIP_2 molecules bind to the L3 site. a) Protocol of the analysis, adapted from Barbera et al (2018). Step 1: PIP_2 molecules which bind to TRPC3 for more than 10 µs were analysed, using gmx mindist. Step 2: From this, we created a binary data array, where a value of 1 indicates a contact between the PIP_2 molecule and a residue of TRPC3 at time t. A value of 0 indicates no contact (we used a threshold of 0.6 nm). Step 3: Next, the Pearson correlation coefficient (PCC) was used to identify residues which

concurrently contact the PIP2 molecule. For example, for lipid x in the figure, the binary array of R374 was compared with the binary array of L375 to calculate the likelihood of concurrent contact with lipid x. This analysis is done pairwise for each residue of TRPC3. Step 4: The results were then collated into an adjacency matrix, where a positive value indicates the two residues are likely to form simultaneous contacts to the bound PIP_2. Step 5: The adjacency matrices of different PIP_2 binding events were then compared statistically using the φ coefficient. The output is a value ranging from -1 to +1. Positive values indicate the two PIP_2 binding events involve the same subset of TRPC3 residues whereas negative values indicate the two PIP_2 binding events do not involve the same subset of TRPC3 residues. This analysis was conducted pairwise for each PIP_2 binding event. In order to create a 2D plot of the φ coefficients, the φ coefficient values were clustered using agglomerative hierarchical clustering according to the single linkage criterion. For more information please see the relevant section in Methods. b) Output of the φ coefficient analysis. Three groups of binding events can be identified, as shown in the panel c. c) Group 2 refers to residues which bind to the L3 site. PIP_2 molecules in group 1 bind to a site adjacent to the L3. PIP_2 molecules in group 3 bind at other sites on TRPC3, such as the L1 binding site.

a)

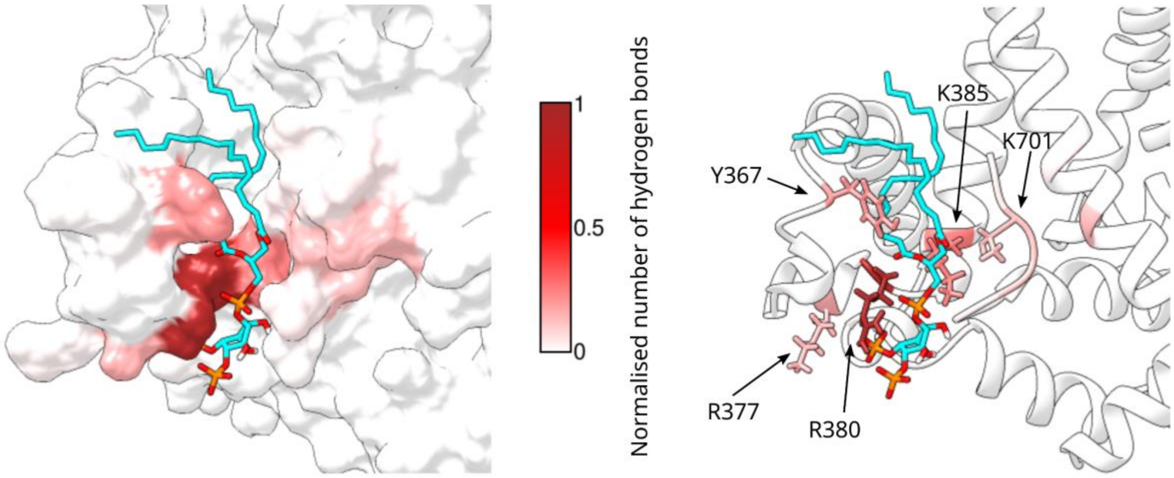

b)

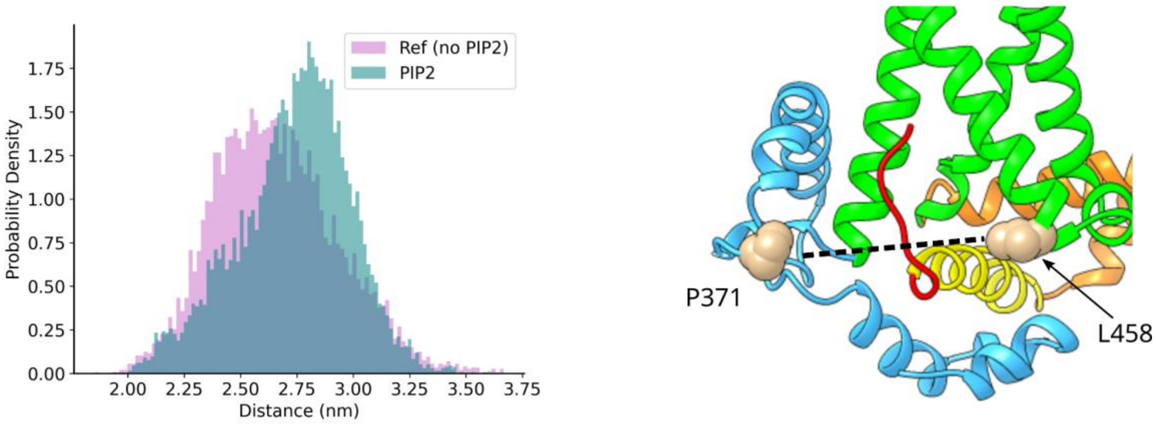

Supplementary Figure 4: All atom simulations reveal local changes to the L3 site in the presence of PIP_2. a) Normalised number of hydrogen bonds between PIP_2 bound at the L3 site and residues of TRPC3. PIP_2 is shown in cyan, with phosphate atoms coloured in orange and oxygen atoms coloured in red. TRPC3 is shown in a (left) surface representation and a (right) ribbon representation. b) Histogram showing the distance between the Cα atoms of P371 (pre-S1 helix) and L458 (S2 helix) residues for the (pink) PIP_2-free, reference membrane, and the (blue) PIP_2 membrane. The residues selected for the distance calculation are shown on the right, with the elements of TRPC3 coloured according to Figure 2e.

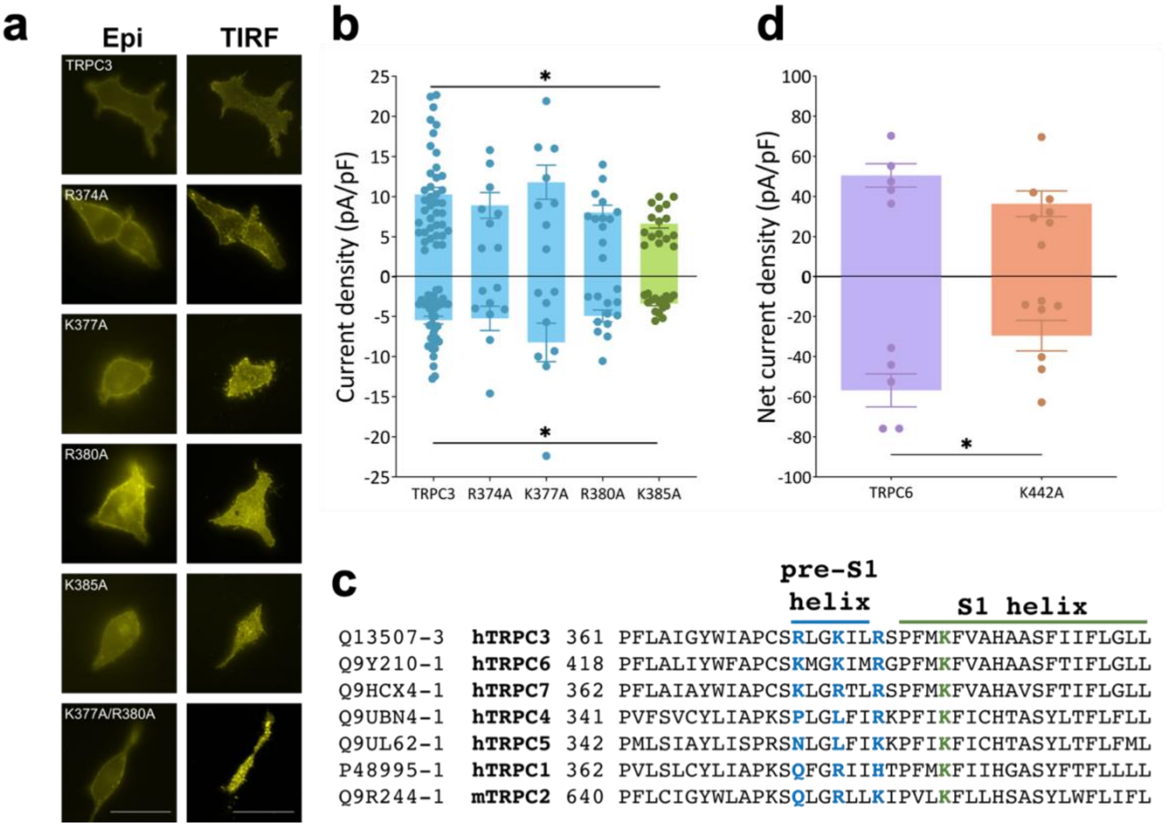

Supplementary Figure 5. The L3 binding site is highly conserved among TRPC channels. a) Epifluorescence and TIRF images of single cells expressing TRPC3 WT and TRPC3 mutants. N = number of cells measured > 5 for each of the constructs. Scale bar is 5 µm. b) Whole-cell basal current density recorded from HEK cells expressing TRPC3 or TRPC3 mutants at membrane potentials of −90 mV and +70 mV. Data presented as Mean±SEM (n = 8-39 cells). Statistical significance was assessed using ANOVA followed by a two-tailed multiple t test with Bonferroni correction. *P< 0.05. If not indicated = n.s. c) Sequence alignments of L3 binding site between human TRPCs and mouse TRPC2. Residues that are homologous to the L3 binding site in TRPC3 are highlighted in all isoforms. d) Whole-cell net current density recorded from HEK cells expressing TRPC6 (lilac) or R442A mutant (orange), in response to repeated applications of GSK (10 µM) at membrane potentials of −90 mV and +70 mV. Data presented as Mean±SEM (n = 5-7 cells). Statistical significance was assessed using a two-tailed t test with Bonferroni correction. *P< 0.05. If not indicated = n.s.

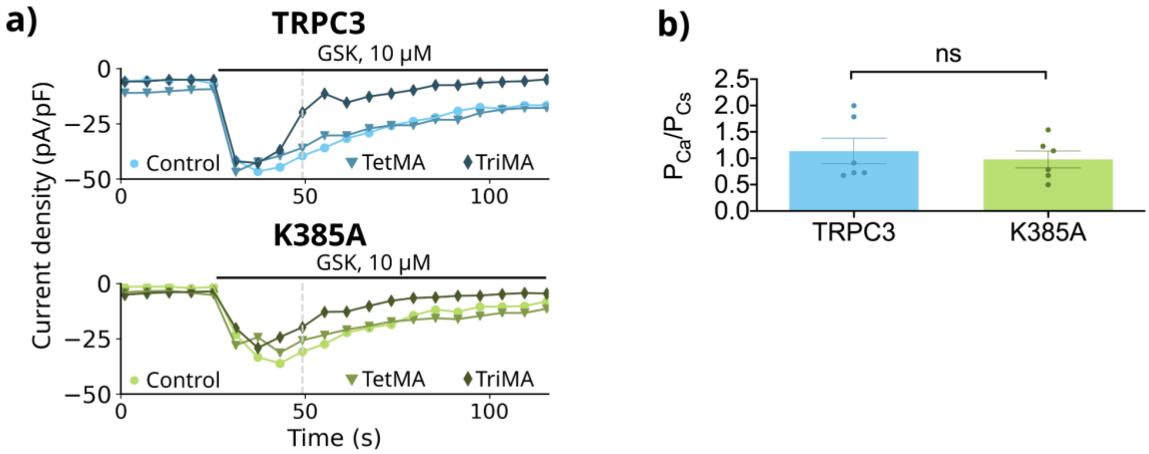

Supplementary Figure 6. The L3 binding site has no impact on the permeation path. a) Representative time course of whole-cell current recorded from HEK cells overexpressing TRPC3 (blue) or K385A mutant (green) in response to repeated applications of GSK (10 µM) at a membrane potential of −90 mV. Dashed lines indicate the switch from standard ECS to solutions in which sodium was equimolarly replaced by the respective amine ($TriMA^+$ or $TetMA^+$). b) Permeability ratio calculated from the reversal potential obtained from HEK cells expressing TRPC3 (blue) or K385A mutant (green) the ECS solution containing 140 mM NMDG and 10 mM Ca^{2+} as the sole charge carrier and pipette ICS containing 145 mM $CsCl_2$ stimulated with GSK (10 µM). Data presented as Mean±SEM (n = 6 cells). Statistical significance was assessed using a two-tailed t test with Bonferroni correction. Not significant - ns.

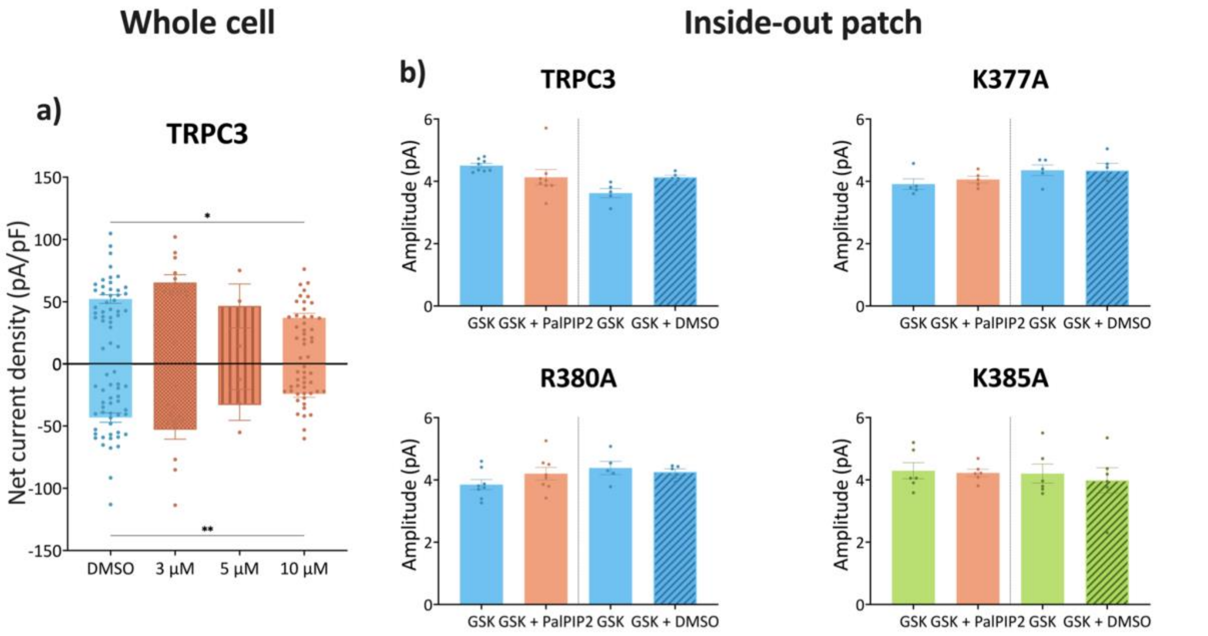

Supplementary Figure 7. Scavenging PIP2 reduces the intensity of the current without affecting the conductance. a) Whole-cell net current density was recorded from HEK cells expressing TRPC3 at membrane potentials of −90 mV and +70 mV. Cells were preincubated with different concentrations of PalPIP2 (3, 5, and 10 µM) for 10 minutes prior to the experiment and then stimulated with GSK (10 µM). Data presented as Mean±SEM (n = 3-36 cells). Statistical significance was assessed using ANOVA followed by a two-tailed multiple t test with Bonferroni correction. *P< 0.05, **P< 0.01. If not indicated = n.s. b) Unitary currents corresponding to open (o) level were recorded from HEK cells expressing TRPC3, K377A, R380A (blue) or K385A mutant (green) in response to repeated applications of GSK (10 µM) or co-application of GSK (10 µM) and PalPIP2 (10 µM; orange) or DMSO (0.01%; patter) simultaneously at a membrane potential of +80 mV. Data obtained in inside-out configuration in gap-free mode and is presented as Mean±SEM (n = 5-8 cells). Statistical significance was assessed using a paired two-tailed t test. If not indicated = n.s.

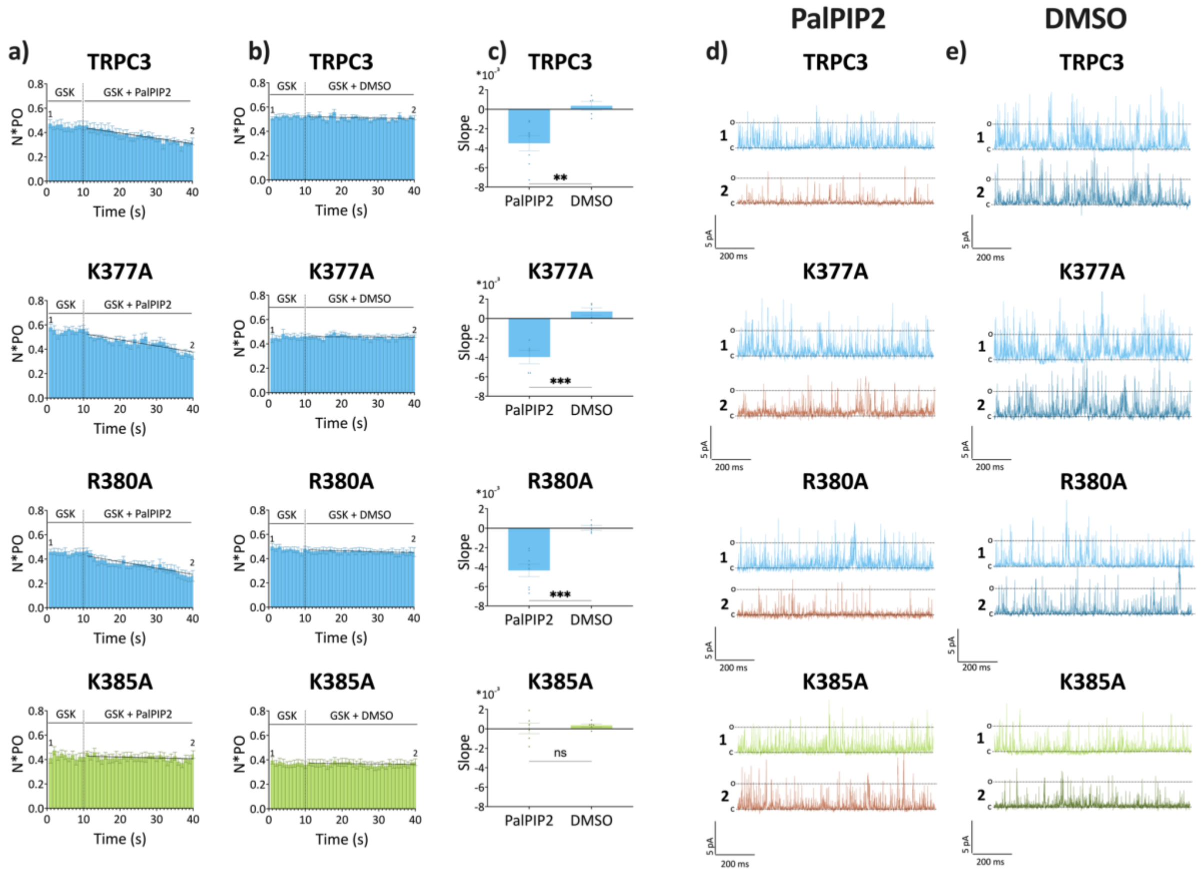

Supplementary Figure 8. The presence of PIP2 at the L3 binding site governs the open probability of TRPC3. Mean values of open probability of TRPC3 or mutants stimulated with GSK (10 µM; TRPC3, K377A, R380A: blue; K385A: green) from 1 to 10 seconds with subsequent co-application of GSK with a) PalPIP2 (10 µM) or b) DMSO (0.01%) from 11 to 40 seconds (the switch of the solutions indicated by the vertical line). Currents recorded at +80 mV in inside-out configuration. Slope of linear regression is shown as a line from 11 till 40 seconds of the recording (after PalPIP2 or DMSO addition) c) Mean values of plotted recordings using simple linear regression of 30 seconds recording (after PalPIP2 or DMSO addition) from cells expressing TRPC3 (PalPIP2, n = 8; DMSO, n = 5; blue), K377A (PalPIP2, n = 5; DMSO, n = 5; blue), R380A (PalPIP2, n = 8; DMSO, n = 5; blue), and K385A (PalPIP2, n = 6; DMSO, n = 6; green). Data presented as Mean±SEM (n = 5-8 cells). Statistical significance was assessed using a two-tailed t test with Bonferroni correction. $**P< 0.01$, $***P< 0.001$. Not significant - ns. Representative single-channel currents show 1 second of d) GSK (10 µM)-induced TRPC3, K377A or R380A (1; blue) or K385A (1; green) recorded at first second of the recording and GSK (10 µM) + PalPIP2 (10 µM)-induced TRPC3, K377A, R380A, and K385A (2; orange) and e) GSK (10 µM)-induced TRPC3, K377A or R380A (1; blue) or K385A (1; green) and GSK (10 µM) + DMSO (0.01%)-induced TRPC3, K377A, and R380A (2; dark blue) or K385A (2; dark green) observed at last second of the recording. Currents recorded at +80 mV in inside-out configuration. Closed (c) and open (o) channel states are indicated.

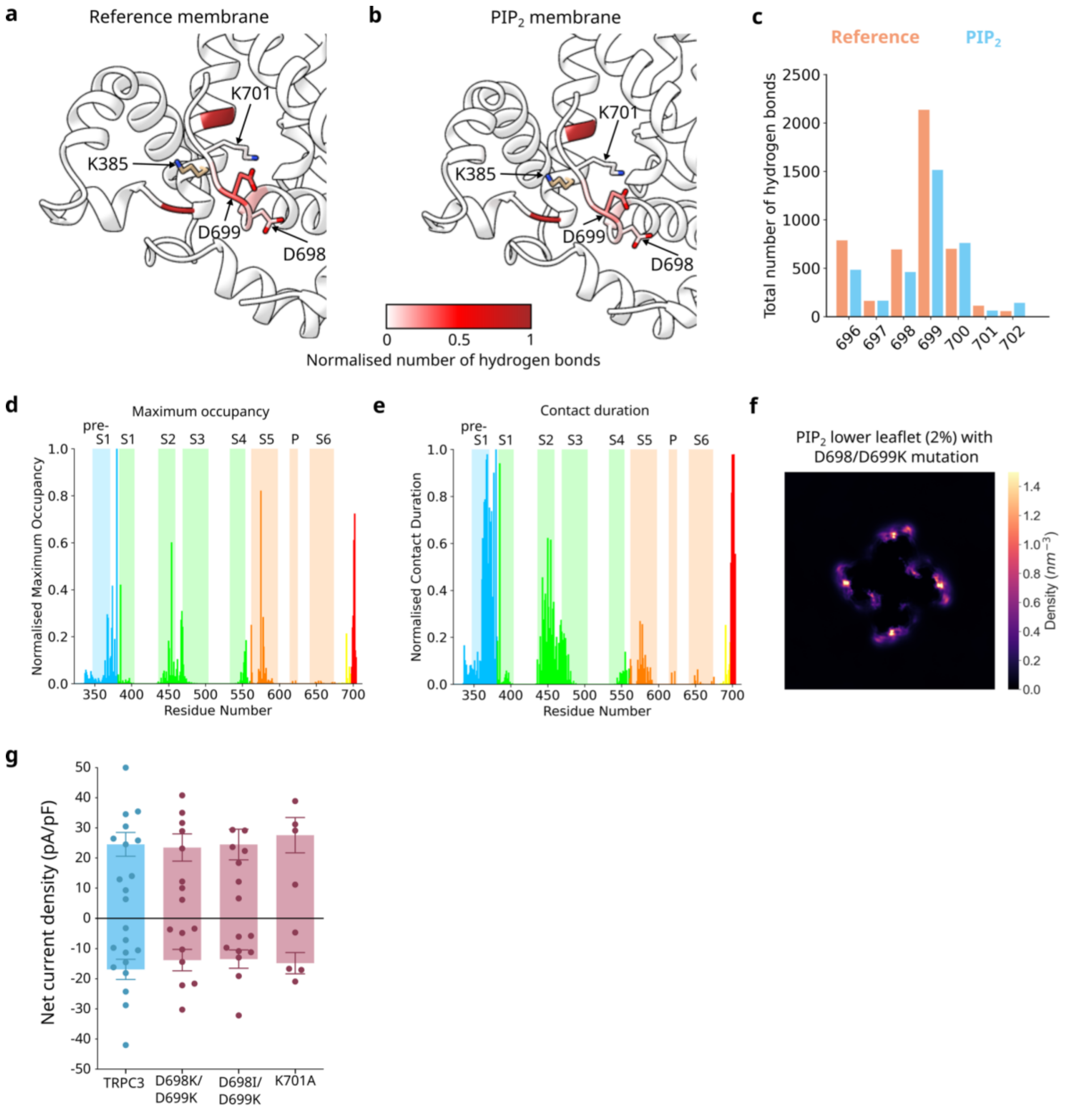

Supplementary Figure 9: K385 interacts with backbones of the residues of the re-entrant loop. a) Normalised number of hydrogen bonds between K385 and residues of TRPC3 in the PIP2-free, reference membrane. b) Normalised number of hydrogen bonds between K385 and residues of TRPC3 in the presence of PIP_2. c) Total number of hydrogen bonds between K385 and residues of the re-entrant loop, in the presence (blue) and absence (beige) of PIP_2. d) Normalised maximum occupancy diagrams for the D698K/D699K mutant interaction with PIP_2. The data is averaged over 20 monomers. The vertical blocks indicate the transmembrane helices of TRPC3, coloured according to (2e). The data was calculated from the equilibrated portion of the simulations (5-20 µs). e) Normalised contact duration for the D698K/D699K mutant interaction with PIP_2. The data is averaged over 20 monomers. The vertical blocks indicate the transmembrane helices of TRPC3, coloured according to (2e). The data was calculated from the equilibrated portion of the simulations (5-20 µs). f) 2D density maps showing PIP_2 density in the lower leaflet of the membrane, at a concentration of 2%, in the presence of the re-entrant loop for the D698K/D699K mutant. g) Whole-cell net current density recorded from HEK cells

expressing TRPC3 or re-entrant loop mutants (D698K/D699K, D698I/D699K and K701A; bordeaux), in response to repeated applications of GSK (10 µM) at a membrane potentials of −90 mV and +70 mV. Data presented as Mean±SEM (n = 4-11 cells). Statistical significance was assessed using ANOVA followed by a two-tailed multiple t test with Bonferroni correction. If not indicated = n.s.

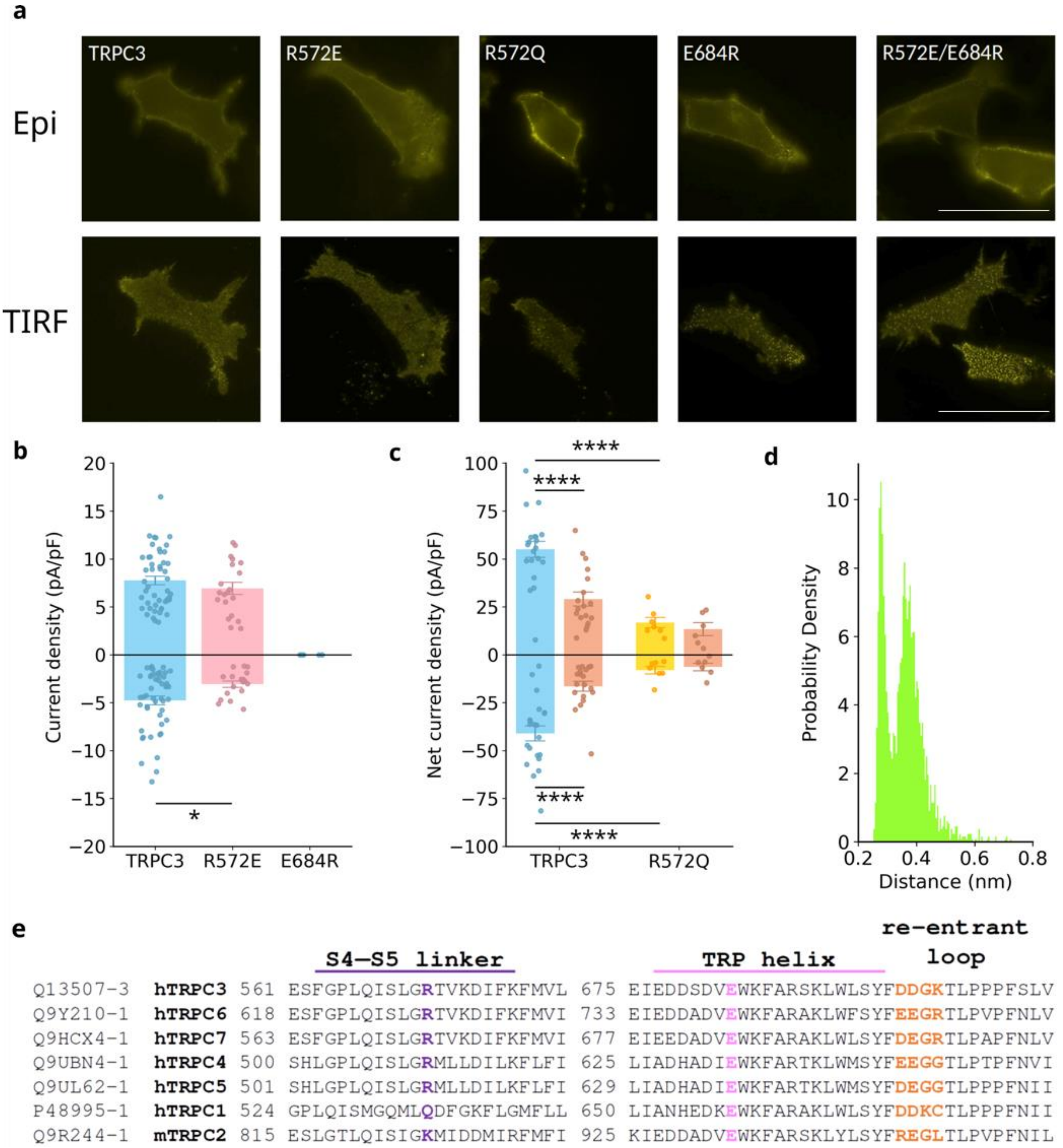

Supplementary Figure 10. a) Epifluorescence and TIRF images of single cells expressing TRPC3 WT and TRPC3 mutants. N = number of cells measured > 5 for each of the constructs. b) Whole-cell basal current density recorded from HEK cells expressing TRPC3 or TRPC3 mutants (R572E - pink, E682R - green) at membrane potentials of −90 mV and +70 mV. Data presented as Mean±SEM (n = 3-48 cells). Statistical significance was assessed using ANOVA followed by a two-tailed multiple t test with Bonferroni correction. *P< 0.05. If not indicated = ns. c) Whole-cell net current density recorded from HEK cells co-expressing TRPC3 or R572Q mutant with either empty mCherry vector (TRPC3 - blue, R572Q - yellow) or mCherry- PH domain (orange), in response to repeated applications of GSK (10 μ M) at a membrane potentials of −90 mV and +70 mV. Data presented as Mean±SEM (n = 6-22 cells). Statistical significance was assessed using a two- tailed t test with Bonferroni correction. ****P<0.0001. If not indicated = ns.

d) Histogram showing the distance between residues in double mutant R572E/E684R, showing a minor recovery of the salt bridge between TRP helix and S4-S5 linker. e) Sequence alignments of the salt bridge forming residues and re-entrant loop in human TRPCs and mouse TRPC2. Residues that are homologous are highlighted in all isoforms.

**Primer**	**Sequence 5´-> 3**
**TRPC3**	
**R374A_hTRPC3_FW**	TTGCAGCGCGCTGGGGAAAATTCTGCGAAGCCCTTTTAT
**R374A_hTRPC3_REV**	CCAGCGCGCTGCAAGGTGCGATCCAGTAGCCAAT
**K377A_Trpc3_FW**	CAGGCTGGGGGCGATTCTGCGAAGCCCTTTTATGAAG
**K377A_Trpc3_REV**	CGCCCCCAGCCTGCTGCAAGGTGCGATCCAG
**R380A_Trpc3_FW**	GGCAAGCCCTTTTATGAAGTTTGTAGCACATGCAGCTTC
**R380A_Trpc3_REV**	CATAAAAGGGCTTGCCAGAATTTTCCCCAGCCTGC
**K385A_hTrpc3_FW**	CCCTTTTATGGCGTTTGTAGCACATG
**K385A_hTrpc3_REV**	CTTCGCAGAATTTTCCCC
**R572E_hTRPC3_FW**	CTCTCTTGGAGAGACTGTAAAGGACATATTCAAGTTC
**R572E_hTRPC3_REV**	ATCTGCAGGGGGCCAAAG
**R572Q_TRPC3_FW**	CTCTTGGACAGACTGTAAAGGACATATTCAAGTTCATGGTCCTC
**R572Q_TRPC3_REV**	TACAGTCTGTCCAAGAGAGATCTGCAGGGGGCCAAAG
**E684R_hTRPC3_FW**	CAGTGATGTACGATGGAAGTTTGCTCG
**E684R_hTRPC3_REV**	TCATCCTCAATTTCTTGATATG
**K701A_hTRPC3_FW**	AACATTACCTCCACCTTTCAGTCTAG
**K701A_hTRPC3_REV**	GCTCCATCATCAAAATAGGATAACCAAAG
**K377A_R380A_Trpc3_FW**	CAGGCTGGGGGCAATTCTGGCAAG
**K377A_R380A_Trpc3_REV**	CTGCAAGGTGCGATCCAG

**TRPC6**	
**K442A_hTRPC6_FW**	GGCGTTTGTAGCACACGCAGCCTCCTTCACCATTTTTCT
**K442A_hTRPC6_REV**	GTGTGCTACAAACGCCATGAATGGTCCACGCATTATCTTC

Supplementary Table 1. Primer sequences.
